# Surgical Left Atrial Appendage Exclusion Does Not Impair Left Atrial Contraction Function: A Pilot Study

**DOI:** 10.1155/2015/318901

**Published:** 2015-07-07

**Authors:** Gijs E. De Maat, Stefano Benussi, Yoran M. Hummel, Sebastien Krul, Alberto Pozzoli, Antoine H. G. Driessen, Massimo A. Mariani, Isabelle C. Van Gelder, Wim-Jan Van Boven, Joris R. de Groot

**Affiliations:** ^1^Department of Cardio-Thoracic Surgery, University Medical Center Groningen, 9711 RB Groningen, Netherlands; ^2^Department of Cardiology, University Medical Center Groningen, 9711 RB Groningen, Netherlands; ^3^Department of Cardiac Surgery, San Raffaele University Hospital, Milan, Italy; ^4^Department of Cardiology, Academic Medical Center, Amsterdam, Netherlands; ^5^Department of Cardio-Thoracic Surgery, Academic Medical Center, Amsterdam, Netherlands

## Abstract

*Background*. In order to reduce stroke risk, left atrial appendage amputation (LAAA) is widely adopted in recent years. The effect of LAAA on left atrial (LA) function remains unknown. The objective of present study was to assess the effect of LAAA on LA function.* Methods*. Sixteen patients with paroxysmal AF underwent thoracoscopic, surgical PVI with LAAA (LAAA group), and were retrospectively matched with 16 patients who underwent the same procedure without LAA amputation (non-LAAA group). To objectify LA function, transthoracic echocardiography with 2D Speckle Tracking was performed before surgery and at 12 months follow-up.* Results*. Mean age was 57 ± 9 years, 84% were male. Baseline characteristics did not differ significantly except for systolic blood pressure (*p* = 0.005). In both groups, the contractile LA function and LA ejection fraction were not significantly reduced. However, the conduit and reservoir function were significantly decreased at follow-up, compared to baseline. The reduction of strain and strain rate was not significantly different between groups.* Conclusions*. In this retrospective, observational matched group comparison with a convenience sample size of 16 patients, findings suggest that LAAA does not impair the contractile LA function when compared to patients in which the appendage was unaddressed. However, the LA conduit and reservoir function are reduced in both the LAAA and non-LAAA group. Our data suggest that the LAA can be removed without late LA functional consequences.

## 1. Introduction

Atrial fibrillation (AF) has a major impact on health care in the Western population and is associated with poor prognosis [1–3]. Besides widely adapted catheter ablation strategies for AF, an emerging treatment strategy is surgical PVI [4, 5]. One of the supposed advantages of this surgical approach is that the LAA can be excluded to reduce stroke risk [6]. Although beneficial effect on morbidity and mortality has not clearly been demonstrated, the LAA is amputated or closed by a clip on a large scale in stand-alone or concomitant AF surgery [7–9]. The effect of this amputation on left atrial function has not been addressed in the current literature.

Current echocardiography guidelines actively recommend the evaluation of LA function after AF to predict the maintenance of sinus rhythm and also identify patients at risk for LA failure or arrhythmias [10]. To assess this left atrial function, recent techniques have been introduced such as two-dimensional speckle tracking echocardiography (2D STE), specifically the parameters strain and strain rate [10, 11]. These novel strain parameters aid to assess the different left atrial functional stages: the reservoir function (storage of PV inflow during ventricular systole), conduit function (passive emptying during early diastole), and contractile function (active emptying at late diastole) [11, 12]. The aim of this study was to investigate the effects of left atrial appendage amputation on left atrial function in the setting of minimally invasive surgical PVI.

## 2. Material and Methods

### 2.1. Patient Population

This observational and retrospective matched group comparison was performed on two series of consecutive patients who were treated for drug resistant paroxysmal AF with sPVI between June 2009 and November 2011 in two centers: Academic Medical Center (AMC), Amsterdam, and University Medical Center Groningen (UMCG) [13]. Patients were matched for gender, age, LA diameter, and AF duration. Inclusion criteria were highly symptomatic paroxysmal AF without concomitant cardiac structural disease, refractory to class I and/or class III AADs or failed catheter ablation for AF. Exclusion criteria for surgical PVI were as follows: left atrial size > 60 mm (parasternal echocardiographic view), prior heart or lung surgery, significant coronary disease or previous MI, left ventricular hypertrophy > 12 mm, previous hospitalization for heart failure, moderate or severe mitral or aortic valve disease, or lung disease (prior tuberculosis or COPD Gold classes III-IV). Furthermore, patients with an ejection fraction < 50% were excluded. Patients in AF or atrial flutter at the time of the echocardiographic analysis were excluded since sinus rhythm is mandatory to reliably objectify the different phases of left atrial function. Definitions of AF, adverse events, and follow-up monitoring were based on the Heart Rhythm Society Consensus Statement for the catheter and surgical ablation of AF [14]. Patients provided written informed consent to the procedure. All patients were treated according to the standard of care for surgical PVI procedures in both AMC and UMCG, and no additional examinations were performed. Clinical and echocardiography data was collected retrospectively and patient privacy was granted by coding of the database according to the rules of good clinical practice and Dutch privacy law. Furthermore, all echocardiography files were anonymized before analysis at the University Medical Center Groningen, Netherlands.

### 2.2. Surgical Procedure

All patients were treated using the video assisted, completely thoracoscopic approach, as detailed previously by Krul et al. [15] and De Maat et al. [13]. In brief, the pulmonary veins were targeted by bilateral thoracoscopy. To isolate the pulmonary veins, a bipolar radiofrequency clamp (Isolator, AtriCure, Inc., Cincinnati, Ohio) was used to create a linear, transmural, thermal lesion. Following the ablation, measurement of effective conduction block was performed by pacing within the PVs (exit block). In the AMC series, also entry block was checked. No additional linear ablations (ablation lines) were applied on the atria. After effective isolation of both right and left PVs, the left atrial appendage was addressed. Concerning the left atrial appendage (LAA) management, in all patients from the LAAA group (AMC) the LAA was amputated with an endoscopic stapling device (Endo Gia stapler, Tyco Healthcare Group, North Haven, CT) [15], whereas in the non-LAAA (UMCG) group the appendage was intentionally not addressed [13].

### 2.3. Echocardiographic Analysis

For this study, a protocol for transthoracic echocardiographic measurement was compiled. In both centers, all patients underwent the echocardiographic analysis performed by experienced sonographers following the protocol. Complete set of measurements are described in Table 2. All images were stored digitally in a DICOM format and stored for offline analysis. Offline analysis was performed by an experienced sonographer (YMH) who was blinded for all other subject characteristics including surgical procedure (LAAA or non-LAAA group). Standard 2D measurements were performed using EchoPac BT12 (General Electric, Horton, Norway); 2D STE software was utilized to analyze LA deformation. All measurements were performed in accordance with the current echocardiographic recommendations and guidelines [10, 11]. Volumetric calculation of both LV and LA was performed using Simpson's biplane method of discs. Additionally LA end systolic volume was indexed to body surface area. Left atrial ejection fraction was calculated as ((LAV_max  _ − LAV_min_)/LAV_max_)*∗*100 [16].

### 2.4. Two-Dimensional Speckle Tracking Echocardiography

**Figure 1:**
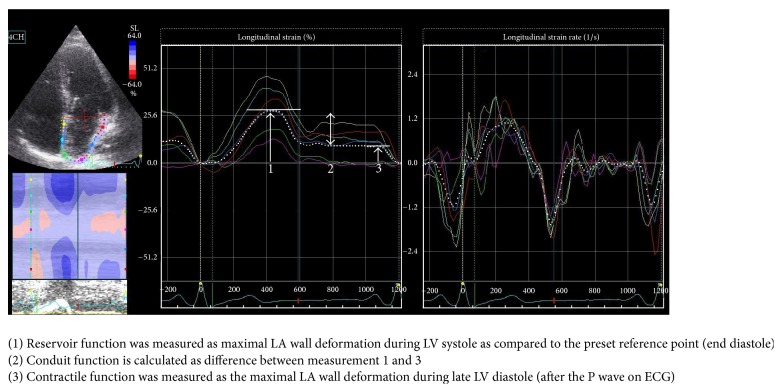
Strain and strain rate measurements.

For the strain measurements, the apical four-chamber view was utilized. As depicted in Figure 1, the edge of the LA endocardium was manually traced after which the software automatically generated tracings based on the speckles of the 2D image. The tracing was all inspected for correctness and manually adjusted if needed and accepted if tracing was acceptable. The software then calculated the mean deformation (strain) expressed in a percentage and speed of deformation (strain rate) expressed as 1/s of the speckles within the region of interest.

As described previously [11, 12], the left atrial function can be divided into three phases. For strain measurements, these are defined as follows: (1)* reservoir function *was calculated as maximal LA wall deformation during LV systole as compared to the preset reference point (end diastole); (2)* conduit function* is considered the maximal LA wall deformation during early LV diastole; and (3)* contractile function* is considered as the maximal LA wall deformation during late LV diastole (after the P wave on ECG). Consecutively, strain rate could be calculated in these three different domains: reservoir, conduit, and contractile function. To determine the effect of the LAAA compared to the non-LAAA group, the delta (before surgery to follow-up difference) of all parameters was compared between groups.

### 2.5. Follow-Up

All patients visited the outpatient clinics according to standard institutional protocol of care for patients treated for AF and underwent a protocolled echocardiogram before surgery and at a median of 12 months (range 6–24 months) after surgery. Periprocedural adverse events were registered. Due to the observational nature of the study, no further specific investigation was requested from the patients.

### 2.6. Endpoints

The primary endpoint was atrial function as evaluated by strain, strain rate, and left atrial ejection fraction, compared between groups. Secondary endpoints were LAAA related adverse events and rhythm outcome at 12-month follow-up without antiarrhythmic drugs.

### 2.7. Statistics

Baseline descriptive statistics are presented as mean ± standard deviation or median (range) for continuous variables, as appropriate, and counts with percentages for categorical variables. Differences between subgroups, in terms of patient characteristics at baseline, different follow-up moments, and end of study, were evaluated by Student's *t*-test or the Mann-Whitney *U* test, depending on normality of the data. Differences within subgroups were evaluated using the Paired *t*-test. Chi-square or Fisher's exact test was used for comparison of categorical variables. The statistical software package IBM SPSS Statistics version 22 was used for all analysis.

## 3. Results

### 3.1. Patient Population

A total of 32 patients were treated with sPVI for lone, drug refractory AF. Mean age was 57 ± 9 years; 84% were male. Paroxysmal AF was present in all 32 patients (100%) and the median AF duration was 3.5 years (range 1–15). In the non-LAAA group 2 (13%) patients had a CHADS2VASC score ≥ 2; in the LAAA group this number was 8 (50%). Two patients underwent previous transcatheter ablation; one patient underwent a cavotricuspid isthmus ablation for a right-sided flutter; another patient underwent transcatheter PVI for AF. The mean systolic and diastolic blood pressure was 134 ± 19 and 82 ± 15 mmHg, respectively. The mean heart rate was 61 ± 10 beats per minute. Baseline clinical patient characteristics did not differ significantly between the two groups except for systolic blood pressure (*p* = 0.005) (Table 1). Before surgery, ventricular diameters, volume, and ejection fraction were similar between groups. The left atrial indexed volume was enlarged with 42 ± 11 in the LAAA group versus 36 ± 5 in the non-LAAA group (*p* = 0.06). Before surgery, strain rate reservoir function differed significantly between groups (*p* = 0.026), but the strain rate conduit and contraction function did not differ between groups (*p* = 0.086 and *p* = 0.079, resp.) (Table 1).

### 3.2. Surgical Treatment

In all patients, the sPVI procedure was completed with proven acute block. Mean procedural time was 160 ± 60 minutes. Mean hospitalization was 7 ± 2 days. The LAA was successfully excluded in all 16 patients in whom LAA amputation was planned. This was objectified by TEE after amputation of the LAA. No periprocedural bleedings and, specifically, no LAAA related bleedings were observed. 

### 3.3. Left Atrial Function

#### 3.3.1. LAAA Group

**Figure 2:**
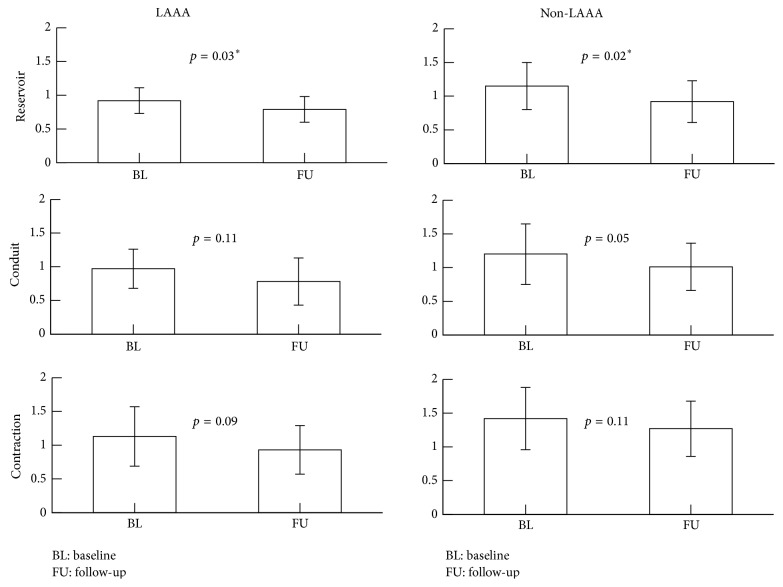
Strain rate bar graph.

At a median of 12-month follow-up echocardiography, the LA diameter and volume indexed to BSA, in the LAAA group, was unchanged compared to baseline measurements (*p* = 0.530, *p* = 0.646, and *p* = 0.735, resp.). Compared to baseline, the strain measured at follow-up of the reservoir and conduit but not contractile phase had decreased (with *p* = 0.007, *p* = 0.014, and *p* = 0.070, resp.). In the strain rate domain, the reservoir function decreased accordingly, but this was not observed in the conduit and contractile phases (with *p* values of 0.029, 0.109, and 0.092, resp.) (Table 2 and Figure 2).

#### 3.3.2. Non-LAAA Group

At follow-up echocardiography, the LA diameter and left atrial volume index of the LAAA group was unchanged compared to baseline measurements (*p* = 0.301, *p* = 0.478, and *p* = 0.773, resp.). Compared to baseline, the strain at follow-up had decreased in reservoir and conduit but not in contractile function (*p* = 0.001, *p* = 0.017, and 0.151, resp.). The strain rate of the reservoir function decreased significantly whereas conduit and contractile function did not, with *p* values of 0.019, 0.053, and 0.108, respectively (Table 2 and Figure 2).

#### 3.3.3. Comparison between Groups

When the delta (difference before surgery and follow-up measurements) is compared between the LAAA and non-LAAA group, none of the changes in atrial dimensions, atrial function, strain, or strain rate measurements differed significantly (Table 2). Left atrial ejection fraction did not differ significantly, neither within nor between groups.

### 3.4. Follow-Up, Procedural Safety, and Rhythm Outcome

Of all patients, pre- and postoperative echocardiography were available and no patients were lost to follow-up. Echocardiography was conducted after a median of 12-month (range 6–24) follow-up. In the LAAA group, no periprocedural (LAA) bleeding occurred. In both investigated groups, 15 (94%) patients were free from atrial arrhythmia and antiarrhythmic medication at 12-month follow-up.

## 4. Discussion

In this retrospective, observational matched group comparison with a convenience sample size of 16 patients, the findings suggest that amputation of the left atrial appendage does not impair the contractile left atrial function or left atrial ejection fraction in patients without structural heart disease. However, the LA reservoir and conduit functions were impaired significantly in both groups at follow-up echocardiography.

Literature does not report the actual contribution of the left atrial appendage to the atrial function or more specifically the contraction. Our study shows that the amputation of the LAA does not significantly affect the left atrial contraction. Our findings contrast with the previous report of Gelsomino et al. who describe improved atrial function and reverse remodeling after successful sPVI [17]. Left atrial volume reduced significantly in these patients, whereas in our group the dimensions did not. This difference might be explained by the more extensive lesion set applied in the series of Gelsomino et al., resulting in more scar related contracture leading to a decrease in left atrial volume [17, 18]. Also, in that series, 49% of patients underwent LAAA or closure; in the other 51% the LAA was not addressed.

It is remarkable that the LA reservoir and conduit function are impaired in both groups following minimally invasive sPVI; this has not been reported previously and might be contributable to the postoperative adhesions of the pericardium and/or antral scarring due to the ablation. Furthermore, it could be hypothesized that this impaired passive left atrial function (conduit and reservoir) precedes active (contractile) left atrial function impairment, as observed in the left ventricle. More research on this topic is warranted.

In our patient population, the rhythm outcome was excellent. This is contributable to our selected, relatively young and healthy patient population with a short history of highly symptomatic paroxysmal atrial fibrillation. Our results are in accordance with the current literature on sPVI procedures [19, 20]. In the present series, no periprocedural bleeding was observed and, specifically, no LAAA related bleedings. This notwithstanding, LAAA related bleedings have been reported, due to the fragile and delicate wall of the LAAA [21, 22].

It has previously been demonstrated that the left atrial appendage is responsible for atrial natriuretic factor (ANF) and performs an important physiologic function regulating the intravascular volume via release of atrial natriuretic peptide. In normal hearts, 30% of the ANF is contained in the LAA. With appropriate medical therapies, postoperative hypertension can be adequately managed, without residual long-term hemodynamic effects.

For this study, we objectified the different left atrial phases by tissue velocity imaging, speckle tracking method. This technique was originally introduced to study left ventricular function. As a spin-off, this technique has been applied to the left atrium and several groups have showed feasibility and good reproducibility in the setting of speckle tracking on the left atrium [11, 23, 24]. As described, this method provides new and interesting information on the left atrial function, specifically reservoir, conduit, and contractile function. However, it remains unclear in what quantities the reservoir, conduit, and contractile phase contribute to the ventricular filling. It also remains unknown what the clinical effect of reduced conduit and reservoir function is, especially in our specific young patient population without major comorbidity. Further study is warranted on the subject of left atrial function, both in healthy subjects and in the context of atrial fibrillation ablation.

## 5. Conclusions

In this retrospective, observational matched group comparison with a convenience sample size of 16 patients, the findings suggest that left atrial appendage amputation does not impair the contractile left atrial function when compared to patients in which the appendage was unaddressed. However, the left atrial conduit and reservoir function decreased in both LAAA and non-LAAA group following minimally invasive surgery for AF. Our data suggest that the LAA can be removed without late left atrial functional consequences.

## Figures and Tables

**Table 1:** Baseline characteristics.

	Non-LAAA group (*n* = 16)	LAAA group (*n* = 16)	*p* value
Age, years	54 ± 10	59 ± 8	0.07
Male, *n* (%)	14 (88%)	13 (81%)	0.63
Median AF history, years [range]	3 [1–10]	4 [1–15]	0.15
AF type			
Paroxysmal, *n* (%)	16 (100%)	16 (100%)	
Previous catheter PVI, *n* (%)	0 (0%)	1 (6%)	0.31
Catheter CTI ablation, *n* (%)	1 (6%)	0 (0%)	0.31
CHA_2_DS_2_-VASC			0.14
0, *n* (%)	7 (44%)	6 (38%)	
1, *n* (%)	7 (44%)	2 (12%)	
≥2, *n* (%)	2 (12%)	8 (50%)	
Body mass index (kg/m²)	28 ± 4	27 ± 3	0.47
Body surface area (m²)	2.15 ± 0.18	2.10 ± 0.23	0.47
Hypertension, *n* (%)	4 (25%)	7 (44%)	0.26
Diabetes, *n* (%)	0 (0%)	1 (6%)	0.31
Stroke history, *n* (%)	0 (0%)	2 (12%)	0.14
Systolic blood pressure (mmHg)	124 ± 19	142 ± 14	<0.01^**∗**^
Diastolic blood pressure (mmHg)	83 ± 18	82 ± 11	0.76
Heart rate (beats/min)	60 ± 9	64 ± 17	0.37
Echocardiography			
LV ejection fraction (%)	59 ± 5	60 ± 7	0.90
LA ejection fraction (%)	40 ± 7	37 ± 10	0.41
LA diameter (mm)	42 ± 6	43 ± 6	0.61
LA volume (mL)	75 ± 15	89 ± 30	0.11
LA volume indexed (mL/m²)	35 ± 5	42 ± 11	0.06
LA strain measurements (%)			
Reservoir function	29.2 ± 7.3	23.1 ± 7.6	0.03^**∗**^
Conduit function	16.1 ± 6.0	12.1 ± 4.9	0.05^**∗**^
Contraction function	13.2 ± 4.8	11.0 ± 5.0	0.22
LA strain rate measurements (s^−1^)			
Reservoir function	1.15 ± 0.35	0.92 ± 0.19	0.03^**∗**^
Conduit function	−1.20 ± 0.45	−0.97 ± 0.29	0.09
Contraction function	−1.42 ± 0.46	−1.13 ± 0.44	0.08

^**∗**^ *p*-value ≤ 0.05.

**Table 2:** Primary endpoints.

Parameter	LAAA group baseline	LAAA group follow-up	*p* value within group	Non-LAAA group baseline	Non-LAAA group follow-up	*p* value within group	Δ between groups
Parasternal LAD (mm)	43 ± 6	43 ± 4	0.53	42 ± 6	44 ± 5	0.30	0.16
LAEF (%)	37 ± 10	37 ± 12	0.92	40 ± 7	37 ± 12	0.64	0.72
LAVI (LAV/BSA)	42 ± 11	41 ± 12	0.74	36 ± 5	36 ± 9	0.77	0.53
Strain reservoir (%)	23.1 ± 7.6	17.1 ± 4.6	<0.01^**∗**^	29.2 ± 7.3	23.6 ± 6.3	<0.01^**∗**^	0.70
Strain conduit (%)	12.1 ± 4.9	8.2 ± 3.5	0.01^**∗**^	16.1 ± 6.0	11.6 ± 5.2	0.02^**∗**^	0.47
Strain contraction (%)	11.0 ± 5.0	8.2 ± 3.3	0.07	13.2 ± 4.8	12.1 ± 4.1	0.15	0.18
Strain rate reservoir (s^−1^)	0.92 ± 0.19	0.79 ± 0.19	0.03^**∗**^	1.15 ± 0.35	0.92 ± 0.31	0.02^**∗**^	0.29
Strain rate conduit (s^−1^)	−0.97 ± 0.29	−0.78 ± 0.35	0.11	−1.20 ± 0.45	−1.01 ± 0.35	0.053	0.94
Strain rate contraction (s^−1^)	−1.13 ± 0.44	−0.93 ± 0.36	0.09	−1.42 ± 0.46	−1.27 ± 0.41	0.11	0.72

BSA = body surface area, LA = left atrium, LAEF = left atrial ejection fraction, LAD = left atrial diameter, PVF = pulmonary vein flow, LAV = left atrial volume, LAVI = left atrial volume indexed, ^*∗*^ *p* value ≤ 0.05.

## Strengths and Limitations

Since a small number of patients were enrolled in this retrospective and observational study, no definite conclusions can be drawn. Although matching provided similar patient characteristics, there were differences between groups at baseline when regarding strain and strain rate. This is counterbalanced by the fact that consistent (echocardiographic) follow-up was conducted and that comparisons are conducted both between and within groups.
